# A new allele of flower color gene *W1 *encoding flavonoid 3'5'-hydroxylase is responsible for light purple flowers in wild soybean *Glycine soja*

**DOI:** 10.1186/1471-2229-10-155

**Published:** 2010-07-28

**Authors:** Ryoji Takahashi, Joseph G Dubouzet, Hisakazu Matsumura, Kentaro Yasuda, Tsukasa Iwashina

**Affiliations:** 1 National Institute of Crop Science, Tsukuba, Ibaraki, 305-8518 Japan; 2 Graduate School of Life and Environmental Sciences, University of Tsukuba, Tsukuba, Ibaraki, 305-8518 Japan; 3 Akita Prefectural University, Ogata, Akita, 010-0451 Japan; 4 Department of Botany, National Museum of Nature and Science, Tsukuba, Ibaraki, 305-0005 Japan; 5 Current Address: Biotransformation Team, Scion Research, Private Bag 3020, Rotorua, New Zealand; 6 Current Address: National Agricultural Research Center for Tohoku Region, Morioka, 020-0198 Japan

## Abstract

**Background:**

*Glycine soja *is a wild relative of soybean that has purple flowers. No flower color variant of *Glycine soja *has been found in the natural habitat.

**Results:**

B09121, an accession with light purple flowers, was discovered in southern Japan. Genetic analysis revealed that the gene responsible for the light purple flowers was allelic to the *W1 *locus encoding flavonoid 3'5'-hydroxylase (F3'5'H). The new allele was designated as *w1-lp*. The dominance relationship of the locus was *W1 *>*w1-lp *>*w1*. One F_2 _plant and four F_3 _plants with purple flowers were generated in the cross between B09121 and a Clark near-isogenic line with *w1 *allele. Flower petals of B09121 contained lower amounts of four major anthocyanins (malvidin 3,5-di-*O*-glucoside, petunidin 3,5-di-*O*-glucoside, delphinidin 3,5-di-*O*-glucoside and delphinidin 3-*O*-glucoside) common in purple flowers and contained small amounts of the 5'-unsubstituted versions of the above anthocyanins, peonidin 3,5-di-*O*-glucoside, cyanidin 3,5-di-*O*-glucoside and cyanidin 3-*O*-glucoside, suggesting that F3'5'H activity was reduced and flavonoid 3'-hydroxylase activity was increased. F3'5'H cDNAs were cloned from Clark and B09121 by RT-PCR. The cDNA of B09121 had a unique base substitution resulting in the substitution of valine with methionine at amino acid position 210. The base substitution was ascertained by dCAPS analysis. The polymorphism associated with the dCAPS markers co-segregated with flower color in the F_2 _population. F_3 _progeny test, and dCAPS and indel analyses suggested that the plants with purple flowers might be due to intragenic recombination and that the 65 bp insertion responsible for gene dysfunction might have been eliminated in such plants.

**Conclusions:**

B09121 may be the first example of a flower color variant found in nature. The light purple flower was controlled by a new allele of the *W1 *locus encoding F3'5'H. The flower petals contained unique anthocyanins not found in soybean and *G. soja*. B09121 may be a useful tool for studies of the structural and functional properties of F3'5'H genes as well as investigations on the role of flower color in relation to adaptation of *G. soja *to natural habitats.

## Background

Soybean (*Glycine max *(L.) Merr.) is believed to have been domesticated in north-eastern China from its wild relative, *Glycine soja *Sieb. & Zucc. [1]. *Glycine soja *is native throughout China, the adjacent area of Russia, Korea, Japan and Taiwan [1]. Flower color of *G. soja *is almost exclusively purple; by contrast, 33% (5,544 out of 16,855) of the soybean accessions in the USDA Soybean Germplasm Collections have white flowers (Dr. R.L. Nelson, personal communication 2006). The reason why *G. soja *almost lacks flower color variants is uncertain [2]. A few white-flowered *G. soja *accessions were reported in a Chinese germplasm collection, but these had high 100-seed weight, strongly suggesting recent outcrossing with *G. max *[2]. One white-flowered plant (PI 424008C) was found in 1998 among the progeny of a purple-flowered *G. soja *accession (PI 424008A) that was originally introduced from South Korea in 1976 [2]. Genetic analysis indicated that the white flower was caused by a recessive allele at the *W1 *locus similar to the white-flowered soybeans [2].

In soybean, six genes (*W1*, *W2*, *W3*, *W4*, *Wm *and *Wp*) primarily control flower color and two genes (*T *and *Td*) control pubescence color [3,4]. The hydroxylation pattern of B-ring in flavonoids plays an important role in the coloration of seed coats, flower and pubescence of soybeans. The B-ring of flavonoids can be hydroxylated at either the 3' position leading to the production of cyanidin-based pigments, or at both the 3' and 5' positions to produce delphinidin-based pigments. Two key enzymes involved in this pathway are flavonoid 3'-hydroxylase (F3'H) and flavonoid 3'5'-hydroxylase (F3'5'H) which are both microsomal cytochrome P450 dependent monooxygenases that require NADPH as a co-factor [5]. Chromatographic experiments suggested that *T *and *W1 *loci are responsible for the formation of flavonoids with 3', 4' and 3', 4', 5' B-ring hydroxylation patterns, respectively [6-8]. Hence, *T *and *W1 *are presumed to encode F3'H and F3'5'H, respectively. The F3'H cDNA was cloned and characterized from a pair of near-isogenic lines (NILs) for the *T *locus, To7B (*TT*, tawny pubescence) and To7G (*tt*, gray pubescence) [9]. Sequence analysis revealed that they differed by a single-base deletion of C in the coding region of To7G. The deletion generated a truncated polypeptide lacking the GGEK consensus sequence of F3'H gene and the heme-binding domain, resulting in non-functional protein.

The *W1 *gene has a pleiotropic effect on flower and hypocotyl color: soybean cultivars with purple/white flowers have purple/green hypocotyls. The soybean F3'5'H gene was cloned from NILs for *W1 *and confirmed that *W1 *encodes F3'5'H and that the gene of white-flowered NILs contained a 65 bp insertion in the coding region [10]. In addition to the F3'5'H protein, a cytochrome b5 is required for full activity of F3'5'H in petunia, and mutation in cytochrome b5 results in a reduction in F3'5'H activity and alteration of anthocyanin amount and composition [11].

The flavonoids in flower petals of soybean have been analyzed [12,13]. The primary components of anthocyanin in purple-flowered cultivars were malvidin 3,5-di-*O*-glucoside, petunidin 3,5-di-*O*-glucoside, delphinidin 3,5-di-*O*-glucoside and delphinidin 3-*O*-glucoside. In addition, eight flavonol glycosides, kaempferol 3-*O*-gentiobioside, kaempferol 3-*O*-rutinoside, kaempferol 3-*O*-glucoside, kaempferol 3-*O*-glycoside, kaempferol 3-*O*-rhamnosylgentiobioside, kaempferol 7-*O*-glucoside, kaempferol 7-*O*-diglucoside and quercetin 3-*O*-gentiobioside, and one dihydroflavonol, aromadendrin 3-*O*-glucoside were identified [12,13]. The sugar component of kaempferol 3-*O*-glycoside could not be determined. No anthocyanins were detected in Clark NILs for *w1 *and *w4*, Clark-*w1 *and Clark-*w4*, and only a trace amount was detected in Clark-*W3w4*. The amount of flavonols and dihydroflavonol in NILs for *w1 *or *w4 *was largely similar to the NILs with purple flowers suggesting that *W1 *and *W4 *affect only anthocyanin biosynthesis. However, the flavonoid composition in flower petals of *G. soja *has not been analyzed so far.

**Figure 1:**
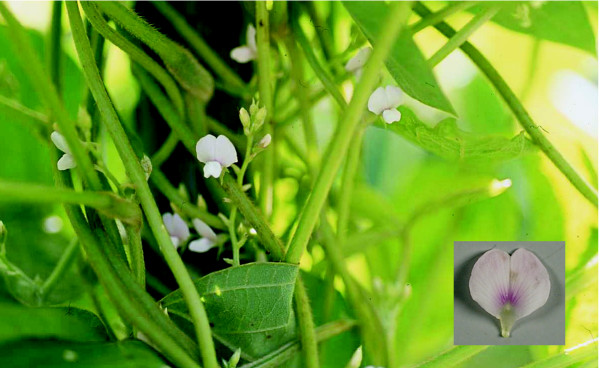
***Glycine soja *line B09121 with light purple flowers discovered in southern Japan**.

Yasuda discovered B09121, a flower color variant of *G. soja*, at a slope between a paddy field and a ditch in Karatsu, Saga Prefecture (southern Japan) in 2002 (unpublished result) (Figure 1). Its banner petals have a pale pinkish hue and a pronounced light purple pigmentation that originates from the base of the petals and spreads in streaks towards the petal margins. We designated the flower color as light purple. Considering its growth habit, small seed size and unique flower color, it is unlikely that the flower color of B09121 was derived from outcrossing with soybean. To our knowledge, B09121 is the first example of a flower color variant of *G. soja *found in nature. The first objective of this study was to determine the genetic basis of purple flower color by crossing experiments. The second objective was to analyze the flavonoids in flower petals of *G. soja *accessions. The third objective was to clone and characterize a gene responsible for light purple flowers.

## Methods

### Genetic analysis

A Canadian soybean cultivar Harosoy (*W1W1 W2W2 w3w3 W4W4 WmWm WpWp tt*) with purple flowers and gray pubescence and a NIL of a US soybean cultivar Clark for *W1 *gene, Clark-*w1 *(L63-2373, *w1w1 W2W2 w3w3 W4W4 WmWm WpWp TT*) with white flowers and tawny pubescence were crossed with B09121 having light purple flowers and tawny pubescence in 2005. Flowers of Harosoy and Clark-*w1 *were emasculated one day before opening and pollinated with B09121. Hybridity of the F_1 _plants was ascertained by tawny pubescence color in crossing with Harosoy and by spindly growth habit in crossing with Clark-*w1*. Seeds of L63-2373 were provided by the USDA Soybean Germplasm Collection. The NIL was produced by crossing Clark with T139 and backcrossing the progeny to Clark up to BC6 [14].

A total of 120 to 130 F_2 _seeds derived from the crossing with Harosoy and Clark-*w1 *were planted in field (low-humic andosols) on June 13 in 2007 at the National Institute of Crop Science, Tsukuba, Japan (36°06'N, 140°05'E). A bulk of 30 seeds each of 36 F_3 _families derived from the cross with Clark-*w1 *were planted at the same location on June 8 in 2008. N, P and K were applied at 3.0, 4.4 and 8.3 g m^-2^, respectively. Flower color was scored in individual F_2 _and F_3 _plants.

### Analysis of flavonoids

Clark, B09121 and four additional *G. soja *accessions were used for flavonoid analysis of flower petals. Seeds of Japanese accessions with purple flowers Kokaigawa-1 collected in Ibaraki Prefecture and COL/AOMORI/1983/NASU-2 collected in Aomori Prefecture were obtained from Ms. N. Mihashi and the NIAS Genebank, respectively. Seeds of a South Korean *G. soja *line with purple flowers (PI 424008A) and its derivative mutant line with white flowers (PI 424008C) were obtained from the USDA Soybean Germplasm Collection. Seeds of the *G. soja *lines were planted in vermiculite in a glasshouse on June 16 in 2006, and transplanted on June 26 to pots (24 cm diameter) filled with 10 kg soil (low-humic andosols). Fertilizers were applied at rates similar to the field plantings.

Banner petals were collected with forceps at the day of opening. Two 200 mg samples of banner petals were collected in 2 ml of MeOH containing 0.1% (v/v) HCl for anthocyanin analysis. Two 200 mg samples in 2 ml of absolute MeOH were also collected for the determination of flavonol and dihydroflavonol. High performance liquid chromatography (HPLC) of anthocyanins, flavonols and dihydroflavonol was performed following previously described protocols [12]. The 2 ml extracts were filtered through disposable filtration units (Maishoridisc H-13-5, Tosoh, Japan) and 10 μl from each sample was subjected to HPLC analysis. The amount of flavonoids was estimated from the pertinent peak area in the HPLC chromatogram (detection wavelength of anthocyanins = 530 nm; flavonols= 351 nm; dihydroflavonols = 290 nm). The peak area was subjected to analysis of variance using Statistica software (StatSoft, Inc. Tulsa, OK).

Three additional anthocyanins were characterized by direct HPLC comparison with authentic specimens, i.e., chrysanthemin (cyanidin 3-*O*-glucoside) from the autumn leaves of *Acer *species [15], cyanin (cyanidin 3,5-di-*O*-glucoside) from the flowers of *Dahlia variabilis *(Willd.) Desf. [16] and peonin (peonidin 3,5-di-*O*-glucoside) from the flowers of *Paeonia albiflora *Pall. var. *hortensis *[17].

### RNA extraction and cDNA cloning

Total RNA was extracted from banner flower petals (100 mg) of Clark, Clark-*w1 *and B09121 using the TRIZOL Reagent (Invitrogen) according to the manufacturer's instructions. cDNA was synthesized by reverse transcription of 5 μg of total RNA using the Superscript III First-Strand Synthesis System (Invitrogen) and an oligo(dT) primer according to the manufacturer's instructions. The full-length cDNA was cloned from Clark and B09121, using a pair of PCR primers (5'-AACTAGCAAATTAATTAGCTT and 5'-CAACCCAAACATTACTTAT) and end-to-end PCR. The PCR mixture contained 0.5 μg of cDNA, 10 pmol of each primer, 10 pmol of nucleotides and 1 unit of ExTaq in 1 × ExTaq Buffer supplied by the manufacturer (Takara) in a total volume of 50 μl. A 5 min denaturation at 94°C was followed by 30 cycles of 30 sec denaturation at 94°C, 1 min annealing at 58°C and 1 min extension at 72°C. A final 7 min extension at 72°C completed the program. The PCR was performed in an Applied Biosystems 9700 thermal cycler. The ~1.8 kbp PCR product was cloned into pCR 2.1 vector (Invitrogen) and sequenced.

### DNA sequencing

Nucleotide sequences of both strands were determined with the BigDye terminator cycle method using an ABI3100 Genetic Analyzer (Applied Biosystems). Nucleotide sequences and the putative amino acid translations were analyzed with the BLAST program [18]. Primers for sequencing the internal regions of F3'5'H genes were 5'-TGTGGTGGTGGAAATGT, 5'-CTATAGAAAGCACCCTTCAA, 5'-GCCTCATCCCTCTTGTTACA and 5'-TTTGAGACGGCGATCCCTTC.

### dCAPS and indel analyses

**Figure 2:**
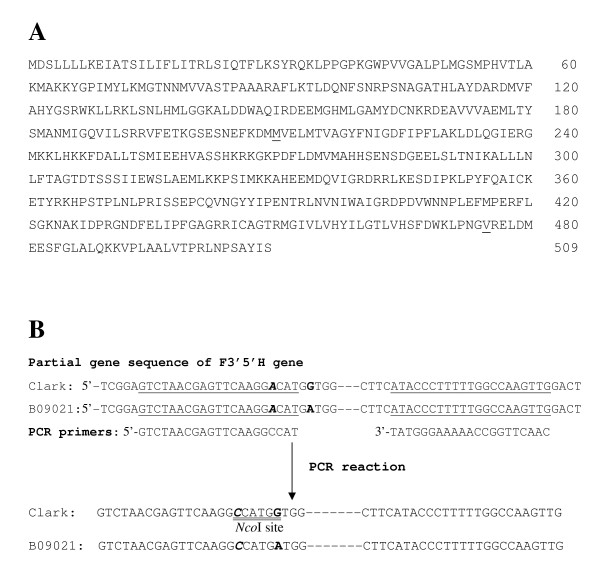
**Amino acid sequence of F3'5'H gene from *Glycine soja* line B09121 with light purple flowers and schematic diagram of dCAPS analysis to detect a base substitution found in B09121.** (A) Amino acid sequence of F3'5'H gene from *Glycine soja *line B09121 with light purple flowers. Amino acid position 210 (underlined) was methionine (valine in Clark) and amino acid position 475 (underlined) was valine (glutamic acid in Clark). (B) Schematic diagram of dCAPS analysis to detect a single-base substitution found in B09121 (shown in bold). *Nco*I site (CCATGG, double underlined) was artificially introduced in the PCR products of Clark using a forward primer with a mismatched base C that is exhibited in bold italic. Annealing sites of PCR primers are underlined. PCR product is digested by *Nco*I only in Clark generating size differences of 18 bp.

Genomic DNA of Clark, Clark-*w1*, B09121 and 36 F_2 _plants that were used for F_3 _progeny tests was isolated from trifoliolate leaves by CTAB [19]. A pair of PCR primers (5'-GTCTAACGAGTTCAAGGCCAT, 5'-CAACTTGGCCAAAAAGGGTAT) was designed to detect a single-base substitution at nucleotide number 653 that is unique to B09121. The first primer contains a nucleotide C that is mismatched with its target DNA to artificially create a restriction site of *Nco*I (CCATGG) in Clark (Figure 2). The base substitution within the restriction site would result in presence/absence of the restriction site in the amplified product to generate a polymorphism. The PCR mixture contained 30 ng of genomic DNA, 5 pmol of each primer, 10 pmol of nucleotides and 1 unit of ExTaq in 1 × ExTaq Buffer supplied by the manufacturer (Takara) in a total volume of 25 μl. After an initial 30 sec denaturation at 94°C, there were 30 cycles of 30 sec denaturation at 94°C, 1 min annealing at 56°C and 1 min extension at 72°C. A final 7 min extension at 72°C completed the program. The amplified products were digested with *Nco*I, and the digests were separated on an 8% nondenaturing polyacrylamide gel in 1 × TBE buffer (90 mM Tris-borate, 2 mM EDTA, pH 8.0). After electrophoresis, the gel was stained with ethidium bromide and the DNA fragments were visualized under UV light.

A pair of indel PCR primers (5'-TTTTGAGCTTATTCCATTTGG, 5'-TGAATATTCGAACCCAACCA) was designed to identify the 65 bp insertion in F3'5'H gene of soybean lines with *w1 *allele based on the previous report [10]. The PCR profile and electrophoresis conditions were identical with the dCAPS analysis except that annealing was conducted at 59°C.

### Semi-quantitative RT-PCR analysis

Semi-quantitative RT-PCR was conducted by reverse-transcription of 5 μg of total RNA using the Superscript III First-Strand Synthesis System and an oligo d(T) primer according to the manufacturer's instruction. To test the transcription level of the F3'5'H gene, PCR reactions were carried out in a volume of 25 μl, using 125 ng of cDNA. The initial 30 sec denaturation at 94°C was followed by 26 cycles of 30 sec denaturation at 94°C, 1 min annealing at 59°C and 1 min extension at 72°C. A final 7 min extension at 72°C completed the program. The primers were 5'-GACGCTGAGGATATTCAACC and 5'-AGAAATCTGTGAGGTCACGA. A soybean actin gene was used as a control. The initial 30 sec denaturation at 94°C was followed by 20 cycles of 30 sec denaturation at 94°C, 1 min annealing at 56°C and 1 min extension at 72°C. A final 7 min extension at 72°C completed the program. The primers were 5'-CTGGGGATGGTGTCAGCCACAC and 5'-CACCGAACTTTCTCTCGGAAGGTG. PCR products were loaded on a 1.2% agarose gel, stained by ethidium bromide and visualized under UV light.

### Accession Numbers

Sequence data from this article have been deposited with the DDBJ Data Libraries under accession nos. AB540111 (Clark) and AB540112 (B09121).

## Results

### Genetic analysis

F_1 _plants produced by a cross between the purple-flowered cultivar Harosoy and B09121 had purple flowers, indicating that purple flower was dominant to light purple flower (Table 1). The F_2 _plants segregated into 3 purple: 1 light purple flower suggesting involvement of a single gene.

**Table 1:** Segregation of flower color in F_1 _plants and F_2 _population derived from a cross between a soybean cultivar Harosoy with purple flowers and B09121, a *Glycine soja *accession with light purple flowers, and segregation of flower color in F_1 _plants, and F_2 _and F_3 _populations derived from a cross between a soybean near-isogenic line Clark-*w1 *with white flowers and B09121 in Tsukuba, Japan.

Generation	Year	Number of plants				Expected ratio	χ^2 ^value	Probability (*P *value)
					
		Total	Purple	Light purple	White			
B09121 (B)	2007	10	-	10	-	-	-	-

Harosoy (H)	2007	10	10	-	-	-	-	-

H × B F_1_	2007	3	3	-	-	-	-	-

H × B F_2_	2007	119	91	28	-	3:1	0.14	0.71

Clark-*w1 *(*w1*)	2007	10	-	-	10	-	-	-

*w1 *× B F_1_	2007	3	-	3	-	-	-	-

*w1 *× B F_2_	2007	124	1	98	25	3:1*^a^*	1.43	0.23

*w1 *× B F_3 _(purple)*^b^*	2008	69	51	18	-	3:1	0.04	0.83

*^a^*Plants with light purple flowers: plants with white flowers.

*^b^*A line derived from an F_2 _plant with purple flowers.

F_1 _plants produced from a cross between Clark-*w1 *and B09121 had light purple flowers (Table 1). Segregation of the F_2 _plants fitted a 3 light purple: 1 white ratio except one plant that had purple flowers. The results suggested that the *W1 *locus was responsible for light purple flowers and that light purple flower was dominant to white flower. Six F_3 _families derived from F_2 _plants with white flowers produced only plants with white flowers (Table 2). Thirty F_3 _families derived from F_2 _plants with light purple flowers produced twenty-one families segregating for flower color and nine families fixed for light purple flowers; the segregation pattern fit a ratio of 2 segregating lines: 1 line fixed for light purple flowers (Table 2). Among the twenty-one segregating families, seventeen families segregated into light purple and white flowers, whereas four families each contained one plant with purple flowers in addition to light purple and white flowers. The frequency of plants with light purple and white flowers fitted 3:1 ratio in the twenty-one segregating families. The frequency of purple-flowered plants was similar across generations; 0.81% (1/124 plants) in F_2 _and 0.95% (4/421 plants) in F_3_. Progeny of an F_2 _plant with purple flowers segregated at a ratio of 3 purple: 1 light purple flower (Table 1). Results of the F_3 _progeny tests supported the hypothesis that light purple flower was controlled by a new allele at the *W1 *locus that was dominant to the *w1 *allele. We designated the allele as *w1-lp *(light purple). The gene symbol was approved by the Soybean Genetics Committee. Dominance relationship of the locus was *W1 *>*w1-lp *>*w1*.

**Table 2:** Segregation of flower color in F_3 _lines derived from a cross between a soybean near-isogenic line Clark-*w1 *with white flowers and B09121, a *Glycine soja *accession with light purple flowers in Tsukuba, Japan in 2008.

Line	Number of lines				Expected ratio	χ^2 ^value	Probability (*P *value)
				
	Total	Fixed for light purple	Segregating	Fixed for white			
F_3 _(light purple)*^a^*	30	9	21*^b^*	-	1:2	0.15	0.70

F_3 _(white)*^c^*	6	-	-	6	-	-	-

*^a^*F_3 _lines derived from F_2 _plants with light purple flowers.

*^b^*Four lines each contained one plant with purple flowers.

*^c^*F_3 _lines derived from F_2 _plants with white flowers.

### Flavonoid analysis

**Figure 3:**
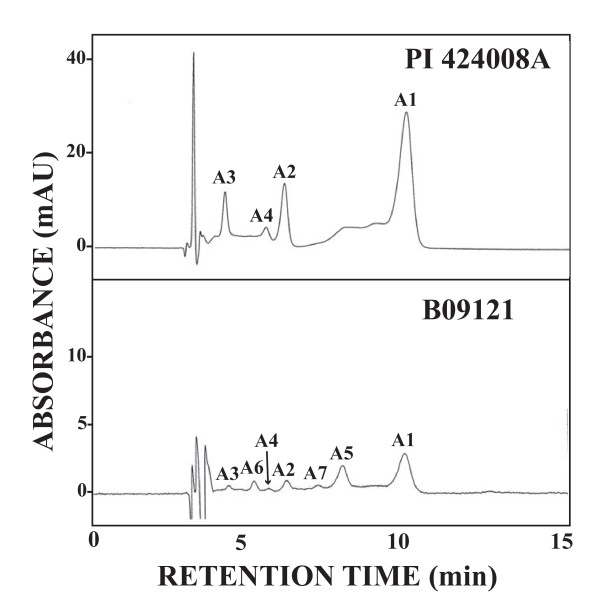
**HPLC chromatogram of anthocyanins extracted from flower petals of PI 424008A (upper panel) and B09121 (lower panel)**. 200 mg of banner petals was extracted with 2 ml of MeOH containing 0.1% HCl. Eluents: MeCN/HOAc/H_2_O/H_3_BO_3 _(6:8:83:3). Flow-rate: 1.0 ml/min. Injection: 10 μl. Detection: 530 nm, A1 = malvidin 3,5-di-*O*-glucoside, A2 = petunidin 3,5-di-*O*-glucoside, A3 = delphinidin 3,5-di-*O*-glucoside, A4 = delphinidin 3-*O*-glucoside, A5 = peonidin 3,5-di-*O*-glucoside, A6 = cyanidin 3,5-di-*O*-glucoside, A7 = cyanidin 3-*O*-glucoside.

The amount of anthocyanins estimated from peak area by HPLC analysis is presented in Table 3. HPLC chromatograms for anthocyanins in PI 424008A and B09121 are presented in Figure 3. In the purple-flowered *G. soja *lines, four HPLC peaks, viz., A1 (malvidin 3,5-di-*O*-glucoside), A2 (petunidin 3,5-di-*O*-glucoside), A3 (delphinidin 3,5-di-*O*-glucoside) and A4 (delphinidin 3-*O*-glucoside), were found similar to Clark. The amount of malvidin 3,5-di-*O*-glucoside in purple-flowered *G. soja *lines was 78 to 252% higher than Clark. The amounts of the other three anthocyanins were not substantially different from Clark.

**Table 3:** Mean and standard errors (× 10^3^) for anthocyanin contents by HPLC analysis of flower petals from soybean cv, Clark and five *G. soja *accessions in 2006 at Tsukuba, Japan.

Line name	A1^a^	A2	A3	A4	A5	A6	A7	Total
Clark	766 ± 186	282 ± 20	399 ± 45	191 ± 10	0 ± 0	0 ± 0	0 ± 0	1,638 ± 260

COL/AOMORI/1983/NASU-2	2,700 ± 769	396 ± 106	315 ± 61	201 ± 23	0 ± 0	0 ± 0	0 ± 0	3,613 ± 959

Kokaigawa-1	1,927 ± 438	340 ± 20	374 ± 17	195 ± 11	0 ± 0	0 ± 0	0 ± 0	2,836 ± 486

PI 424008A	1,365 ± 228	350 ± 24	366 ± 9	196 ± 21	0 ± 0	0 ± 0	0 ± 0	2,276 ± 174

PI 424008C	0 ± 0	0 ± 0	0 ± 0	0 ± 0	0 ± 0	0 ± 0	0 ± 0	0 ± 0

B09121	317 ± 4	172 ± 4	147 ± 2	116 ± 2	287 ± 19	160 ± 1	142 ± 18	1,342 ± 33

LSD_0.05_	1,384	166	116	52	29	2	27	1,662

^a^A1: malvidin 3,5-di-*O*-glucoside, A2: petunidin 3,5-di-*O*-glucoside, A3: delphinidin 3,5-di-*O*-glucoside, A4: delphinidin 3-*O*-glucoside, A5: peonidin 3,5-di-*O*-glucoside, A6: cyanidin 3,5-di-*O*-glucoside, A7: cyanidin 3-*O*-glucoside.

In contrast, flower petals of white-flowered line PI 424008C contained no anthocyanins. Further, the flower petals of B09121 contained about half the amount of A1 to A4 compared with Clark. The HPLC chromatogram of B09121 exhibited three additional anthocyanin peaks, A5 to A7, that were not found in soybeans or other *G. soja *lines (Figure 3). Based on the comparison of retention time with authentic specimens, peonin, cyanin and chrysanthemin, A5, A6 and A7 were estimated as peonidin 3,5-di-*O*-glucoside, cyanidin 3,5-di-*O*-glucoside and cyanidin 3-*O*-glucoside, respectively.

Eight peaks, F1 to F8, corresponding to flavonol glycosides, were detected by HPLC analysis: F1 (kaempferol 3-*O*-gentiobioside), F2 (kaempferol 3-*O*-rutinoside), F3 (kaempferol 3-*O*-glucoside), F4 (kaempferol 3-*O*-glycoside), F5 (kaempferol 3-*O*-rhamnosyl-(1→2)-[glucosyl-(1→6)-galactoside]), F6 (quercetin 3-*O*-gentiobioside), F7 (kaempferol 7-*O*-glucoside) and F8 (kaempferol 7-*O*-diglucoside). The sugar component of F4 could not be determined. Though F5 has been partially characterized as kaempferol 3-*O*-rhamnosylgentiobioside [12], our data indicates that it is actually kaempferol 3-*O*-rhamnopyranosyl-(1→2)-[glucopyranosyl-(1→6)-galactopyranoside] by ^1^H and ^13^C NMR survey. The pertinant ^1^H and ^13^C NMR data of F5 are as follows:

^1^H NMR (600 MHz, pyridine-*d*_5_): δ 13.43 (1 H, s, 5-OH), 8.61 (2 H, d, *J *= 8.8 Hz, H-2',6'), 7.30 (2 H, d, *J *= 8.9 Hz, H-3',5'), 6.80 (1 H, d, *J *= 2.1 Hz, H-8), 6.72 (1 H, d, *J *= 2.0 Hz, H-6), 6.39 (1 H, d, *J *= 7.7 Hz, galactosyl H-1), 6.34 (1 H, s, rhamnosyl H-1), 4.73 (1 H, d, *J *= 7.8 Hz, glucosyl H-1), 1.53 (3 H, d, *J *= 6.2 Hz, rhamnosyl CH_3_). ^13^C NMR (125 MHz, pyridine-*d*_5_): (kaempferol) δ 157.4 (C-2), 134.2 (C-3), 178.7 (C-4), 163.0 (C-5), 99.7 (C-6), 165.7 (C-7), 94.5 (C-8), 157.6 (C-9), 105.5 (C-10), 122.3 (C-1'), 131.9 (C-2',6'), 116.2 (C-3',5'), 161.6 (C-4'); (galactose) δ 100.7 (C-1), 76.5 (C-2), 75.7 (C-3), 69.5 (C-4), 74.9 (C-5), 68.3 (C-6); (glucose) δ 104.9 (C-1), 75.0 (C-2), 78.1 (C-3), 71.4 (C-4), 78.3 (C-5), 62.3 (C-6); (rhamnose) δ 102.5 (C-1), 72.9 (C-2), 72.7 (C-3), 74.2 (C-4), 69.9 (C-5), 18.3 (C-6).

The amounts of flavonol glycosides estimated by peak areas in HPLC analysis are presented in Table 4. PI 424008A and PI 424008C contained all eight of the flavonol glycosides found in Clark. COL/AOMORI/1983/NASU-2 lacked F4 and Kokaigawa-1 lacked F8. B09121 was devoid of F4 and F7. However, the total amount of flavonol glycosides was not significantly different among the Clark and *G. soja *lines included in this report.

**Table 4:** Mean and standard errors (× 10^3^) of flavonol contents by HPLC analysis of flower petals from soybean cv, Clark and five *G. soja *accessions in 2006 at Tsukuba, Japan.

Line name	F1^a^	F2	F3	F4	F5	F6	F7	F8	Total
Clark	7,581 ± 391	792 ± 160	221 ± 78	300 ± 24	323 ± 45	162 ± 36	88 ± 60	144 ± 35	9,612 ± 829

COL/AOMORI/1983/NASU-2	11,097 ± 357	816 ± 40	256 ± 7	0 ± 0	724 ± 40	177 ± 17	36 ± 2	240 ± 1	13,346 ± 463

Kokaigawa-1	8,812 ± 611	56 ± 3	159 ± 13	327 ± 27	215 ± 17	207 ± 35	29 ± 6	0 ± 0	9,805 ± 712

PI 424008A	14,138 ± 4,800	1,091 ± 328	339 ± 102	624 ± 226	643 ± 196	290 ± 124	156 ± 53	199 ± 65	17,481 ± 5,896

PI 424008C	14,025 ± 637	774 ± 3	321 ± 20	533 ± 5	585 ± 36	116 ± 3	29 ± 1	131 ± 28	16,513 ± 686

B09121	13,465 ± 1,026	776 ± 92	279 ± 27	0 ± 0	745 ± 61	376 ± 7	0 ± 0	228 ± 27	15,868 ± 1,240

LSD_0.05_	ns^b^	562	ns	340	323	ns	ns	124	ns

^a^F1: kaempferol 3-*O*-gentiobioside, F2: kaempferol 3-*O*-rutinoside, F3: kaempferol 3-*O*-glucoside, F4: kaempferol 3-*O*-glycoside, F5: kaempferol 3-*O*-rhamnosyl-(1→2)-[glucosyl-(1→6)-galactoside], F6: quercetin 3-*O*-gentiobioside, F7: kaempferol 7-*O*-glucoside, F8: kaempferol 7-*O*-diglucoside.

^b^not significant at 5% level by analysis of variance

One peak (F9) corresponding to dihydroflavonol (aromadendrin 3-*O*-glucoside) was found by HPLC analysis in Clark and all of the *G. soja *lines. The amount of aromadendrin 3-*O*-glucoside estimated by peak area in HPLC analysis is presented in Table 5. The *G. soja *lines had 33 to 155% more aromadendrin 3-*O*-glucoside than Clark.

**Table 5:** Mean and standard errors (× 10^3^) of dihydroflavonol contents by HPLC analysis of flower petals from soybean cv, Clark and five *G. soja *accessions in 2006 at Tsukuba, Japan.

Line name	F9^a^
Clark	789 ± 54

COL/AOMORI/1983/NASU-2	1,286 ± 38

Kokaigawa-1	1,046 ± 53

PI 424008A	1,574 ± 382

PI 424008C	1,547 ± 91

B09121	2,010 ± 94

LSD_0.05_	612

^a^F9: aromadendrin 3-*O*-glucoside

### cDNA cloning

Four F3'5'H cDNA clones each from Clark and B09121 were sequenced from the 5' and 3' ends. The cDNA sequence of both lines consisted of 1657 nucleotides encoding 509 amino acids (Figure 2). Compared with Clark, five nucleotides were substituted in B09121 at nucleotide positions 653, 1084, 1366, 1449 and 1534 in the coding region. Identical base substitutions at nucleotide positions 1084, 1449 and 1534 were also observed in cultivar Chin-Ren-Woo-Dou with purple flowers (GenBank accession number AY117551). Substitution at nucleotide positions 1084, 1366 and 1534 had no effects on amino acid sequences. In contrast, the base substitution at nucleotide positions 653 and 1449 changed amino acids from valine to methionine (amino acid position 210) and from glutamic acid to valine (amino acid position 475), respectively (Figure 2). The latter amino acid substitution was also found in Chin-Ren-Woo-Dou, whereas the former was unique to B09121.

### dCAPS and indel analysis

**Figure 4:**
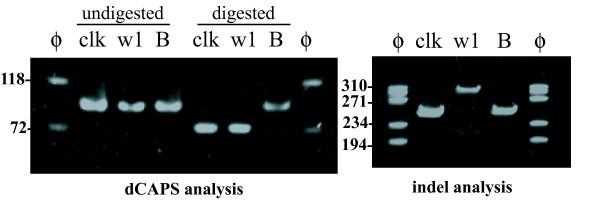
**Results of dCAPS and indel analysis of F3'5'H gene in a soybean cultivar Clark, a near-isogenic line of Clark with white flowers, Clark-*w1 *and a *Glycine soja *line with light purple flowers, B09121**. (Left panel) Results of dCAPS analysis. PCR products amplified with dCAPS primers were digested by *Nco*I and the digests were separated on an 8% polyacrylamide gel. ϕ, molecular marker ϕx174/*Hae*III; clk, Clark; w1, Clark-*w1*; B, B09121. The migration of size markers is shown to the left of the gel. (Right panel) Results of indel analysis. PCR products amplified with indel primers were separated on an 8% polyacrylamide gel. ϕ, molecular marker ϕx174/*Hae*III; clk, Clark; w1, Clark-*w1*; B, B09121.

The PCR reaction for dCAPS analysis produced bands with expected size of about 100 bp in Clark, Clark-*w1 *and B09121 (Figure 4). *Nco*I digested the bands of Clark and Clark-*w1 *and shortened the bands by 18 bp. In contrast, the band from B09121 was largely undigested. In addition, a faint band with approximately the same size was also observed among the digested bands in B09121. Similar results were obtained in dCAPS analysis using a different set of PCR primers and a different restriction enzyme (*Hph*I) (data not shown). The PCR reaction for indel analysis produced shorter bands (255 bp) in Clark and B09121, and a longer band in Clark-*w1 *(310 bp) due to the 65 bp insertion (Figure 4). In addition, a faint band was also observed in Clark-*w1 *that was approximately the same size as the shorter bands in Clark and B09121.

**Figure 5:**
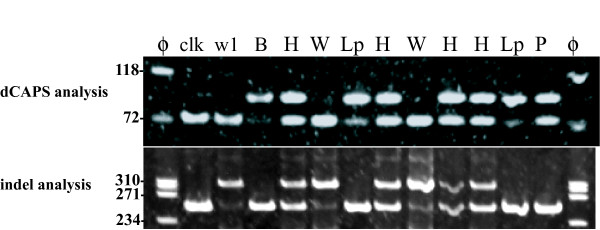
**dCAPS and indel analyses of an F_2 _population derived from a cross between a soybean near-isogenic line with white flowers, Clark-*w1 *and a *Glycine soja *line with light purple flowers, B09121**. (Upper panel) Results of dCAPS analysis. PCR products with dCAPS primers were digested with *Nco*I and the digests were separated on an 8% polyacrylamide gel. ϕ, molecular marker ϕx174/*Hae*III; clk, Clark; w1, Clark-*w1*; B, B09121; W, F_2 _plants homozygous for *w1 *allele (white flower); Lp, F_2 _plants homozygous for *w1-lp *allele (light purple flower); H, heterozygous F_2 _plants (light purple flower); P, an F_2 _plant with purple flowers. The migration of size markers is shown to the left of the gel. (Lower panel) Results of indel analysis. PCR products amplified with indel primers were separated on an 8% polyacrylamide gel.

In dCAPS analysis of the F_2 _population, plants with *w1w1 *genotype (white flower) had only shorter bands, whereas plants with *w1-lpw1-lp *genotype (light purple flower) had longer bands with faint bands similar to B09121 (Figure 5). Heterozygous plants with the *w1-lpw1 *genotype (light purple flower) had both bands at similar band intensity. In indel analysis, plants with *w1-lpw1-lp *genotype had only shorter bands, whereas plants with *w1w1 *genotype had longer bands and faint shorter bands. Heterozygous plants with *w1-lpw1 *genotype had both bands at similar band intensity. Thus, dCAPS and indel markers co-segregated in plants with white and light purple flowers. In contrast, the F_2 _plant with purple flowers had the two bands with similar intensities in dCAPS analysis and only a shorter band in indel analysis.

### Semi-quantitative RT-PCR analysis

Results of semi-quantitative RT-PCR suggested that the transcript level of the F3'5'H gene was not substantially different among lines (data not shown).

## Discussion

Flower color of *G. soja *is almost exclusively purple, whereas about 30% of soybean cultivars have white flowers. The reason why *G. soja *almost lacks flower color variants is uncertain [2]. In 1998, researchers found a white-flowered variant of PI 424008A, a USDA accession of *G. soja *with purple flowers that was originally introduced from South Korea in 1976 [2]. The mutation may have occurred during propagation at USDA. To our knowledge, B09121 is the first example of flower color variant found in the natural habitat.

Genetic analysis suggested that light purple flower is controlled by a new allele at the *W1 *locus, *w1-lp*. Dominance relationship of the locus was *W1 *>*w1-lp *>*w1*. Interestingly, one F_2 _plant and four F_3 _plants with purple flowers were generated in the cross with Clark-*w1*. Considering the fact that purple-flowered plants were produced from heterozygous plants (*w1-lp w1*) in both F_2 _and F_3 _generations and that frequency of purple-flowered plants was similar (about 1%) across generations, the purple flower color may have been produced by a crossover in the *W1 *gene instead of seed contamination or out-crossing. The purple-flowered F_2 _plant produced F_3 _plants with purple and light purple flowers at a 3:1 ratio; this suggests that the region including the 65 bp insertion was eliminated from the genome. The dCAPS and indel analyses indicated that the base substitution was heterozygous but the indel region was homozygous without the 65 bp insertion in the purple-flowered F_2 _plant. The results further supported elimination of the insertion that is responsible for gene dysfunction from the plant by intragenic recombination. It remains to be investigated whether the existence of tandem repeats derived from the insertion is responsible for the high frequency of intragenic recombination.

Sequence analysis of F3'5'H cDNA from Clark and B09121 indicated that two amino acids (amino acid numbers 210 and 475) were substituted. The former substitution has not been observed in soybean cultivars examined to date. It may be responsible for light purple flower and unique anthocyanin composition. However, no catalytic domains have been assigned to the region. The spontaneous mutation leading to flower color change may not have affected amount of F3'5'H gene transcripts, based on the results from semi-quantitative RT-PCR analyses.

Flavonoids in flower petals of *G. soja *with purple flowers were generally similar to those of soybean cultivars with purple flowers. Flower petals of PI 424008C with white flowers had no anthocyanin but contained comparable amounts of flavonol glycosides and dihydroflavonols. It is consistent with the result from Clark-*w1*, a soybean NIL with white flowers, whose flower petals contained no anthocyanin although it had levels of flavonol glycosides and dihydroflavonol similar to Clark [12]. The present results confirmed that *W1 *solely controls anthocyanin biosynthesis in *G. soja*.

Purple flowers of soybean and *G. soja *contain four major anthocyanins with 3'4'5'-substituted form, malvidin 3,5-di-*O*-glucoside, petunidin 3,5-di-*O*-glucoside, delphinidin 3,5-di-*O*-glucoside and delphinidin 3-*O*-glucoside. In contrast, flower petals of B09121 contained lower amounts of the four major anthocyanins. In addition, they contained small amounts of the 5'-unsubstituted versions of the above anthocyanins, peonidin 3,5-di-*O*-glucoside, cyanidin 3,5-di-*O*-glucoside and cyanidin 3-*O*-glucoside. It appears that F3'5'H activity was reduced and F3'H activity was increased in flower petals of B09121. Flower petals of soybean and *G. soja *contain large amounts of kaempferols and very small amounts of quercetins [12,13], suggesting that F3'H activity may be very low. Further, alleles at the *T *locus encoding F3'H did not affect 3'-hydroxylation of flavonols in flower petals [12,13]. Therefore, it is unlikely that the F3'H gene might be responsible for the anthocyanin alteration. Alternatively, mutation in the F3'5'H gene possibly led to a reduction in F3'5'H activity and an increase in F3'H activity. In petunia, a cytochrome b5 is required for full activity of F3'5'H. A mutation in cytochrome b5 reduced 3'4'5'-substituted anthocyanins and increased 3'4'-substituted anthocyanins [11]. It is possible that the amino acid substitution might directly affect the amount and composition of anthocyanins or interact with cytochrome b5. Functional analysis using yeast recombinant assays may be necessary to identify the amino acid substitution that led to light purple flower and unique anthocyanin composition, to investigate the association with cytochrome b5, and to verify whether the amino acid substitution generated F3'H activity. Transformation experiments using a soybean line with *w1 *allele may be necessary to verify the function of the F3'5'H gene from B09121. B09121 may be the first example of a flower color variant of *G. soja *found in the natural habitat. It may be a useful tool for studies of the structural and functional properties of F3'5'H genes as well as investigations on the role of flower color in relation to adaptation of *G. soja *to natural habitats.

## Conclusions

This study is the first report of a flower color variant of wild soybean *G. soja *discovered in nature. Genetic analysis revealed that light purple flower of the accession B09121 was controlled by a new allele of *W1 *locus encoding F3'5'H. The new allele was designated as *w1-lp*. The dominance relationship of the locus was *W1 *>*w1-lp *>*w1*. In crossing experiments, purple-flowered plants were generated in the cross between B09121 and a soybean near-isogenic line with *w1 *allele. F_3 _progeny test, and dCAPS and indel analyses suggested that the plants with purple flowers might be due to intragenic recombination. Flower petals of B09121 contained lower amounts of four major anthocyanins common in purple flowers and contained small amounts of the 5'-unsubstituted versions of the above anthocyanins that are absent in soybeans and other *G. soja *accessions. The results suggested that F3'5'H activity was reduced and flavonoid 3'-hydroxylase activity was increased in the flower petals. The cDNA of B09121 had a unique base substitution resulting in the substitution of valine with methionine. B09121 may be a useful tool for studies of the structural and functional properties of F3'5'H genes as well as investigations on the role of flower color in relation to adaptation of *G. soja *to natural habitats.

## Authors' contributions

RT carried out genetic analysis. RT, JGD and HM participated in the molecular genetic studies. KY discovered a *G. soja *line with unique flower color. TI carried out chemical analysis of flavonoids. JGD critically revised the manuscript. All authors read and approved the final manuscript.
